# Measuring general sense of agency: a Japanese adaptation and validation of the sense of agency scale (J-SoAS)

**DOI:** 10.3389/fpsyg.2024.1427169

**Published:** 2024-09-04

**Authors:** Wenzhen Xu, Roberto Legaspi, Yuichi Ishikawa, Yuichi Washida

**Affiliations:** ^1^Graduate School of Business Administration, Hitotsubashi University, Tokyo, Japan; ^2^Human-Centered AI Labs, KDDI Research, Inc., Fujimino, Japan; ^3^Graduate School of Information Science and Electrical Engineering, Kyushu University, Fukuoka, Japan

**Keywords:** sense of agency, scale development and adaptation, reliability, validity, construct stability, age difference

## Abstract

The Sense of Agency (SoA) refers to the individual’s perception of control over actions and their subsequent impact on the external environment. SoA encompasses multiple dimensions, such as implicit/local and explicit/general, which can be quantitatively assessed through cognitive tasks and psychometric questionnaires, respectively. The explicit and general aspect of SoA is commonly evaluated using the Sense of Agency Scale (SoAS). This study’s objective is to adapt and validate a Japanese version of the Tapal-SoAS. To achieve this, we distributed an online survey in three stages, gathering data from 8,237 Japanese participants aged between their 20s and 60s. Our analysis confirmed the bifactorial structure identified in the original study: the Sense of Positive Agency (SoPA) and the Sense of Negative Agency (SoNA). Metrics pertaining to test–retest reliability, internal consistency, and construct validity reached satisfactory thresholds. Furthermore, the two-factor models demonstrated suitable fit across various age cohorts. The Japanese version of the SoAS (J-SoAS) shows potential for cross-cultural comparisons of explicit and general SoA, particularly between Western and Eastern populations, and among distinct age groups, including young adults and the elderly.

## 1 Introduction

### 1.1 What is sense of agency and why it is important?

The Sense of Agency (SoA), a fundamental construct in psychology, is defined as the subjective sensation of control over one’s intentional actions and their consequent effects on the external environment (Gallagher, 2000; Gallagher, 2012; Moore, 2016; Haggard, 2017). SoA emerges when individuals exert control over their actions, influencing their surroundings and subsequently assuming responsibility for the outcomes of their actions (Wen and Imamizu, 2022).

A wealth of literature suggests that the development of SoA commences in early stages of life, shaping individuals’ perceptions and behaviors across their lifespan (Blakemore et al., 1998; Salomon et al., 2013; Wen and Haggard, 2018). For instance, the perceived link between an infant’s movements and the corresponding visual or auditory feedback reinforces motor skills during early developmental stages (Rovee and Rovee, 1969; Siqueland and Delucia, 1969; Rochat, 1998). The sense of control over life events typically diminishes in older adulthood, emphasizing the relevance of maintaining an appropriate level of SoA for the quality of life among elderly individuals (Metcalfe et al., 2010; Cioffi et al., 2017).

SoA also underpins experiences of volition and free will (Haggard, 2017), the acknowledgment of social responsibility for one’s actions (Haggard and Tsakiris, 2009), and the comprehension of causal structures in the world (Legaspi et al., 2021). Moreover, a loss of SoA has been identified as a significant factor in neurological and psychiatric disorders (Moore and Fletcher, 2012; Moore, 2016), as well as poor physical and mental health outcomes (Haggard, 2017), thereby bearing substantial implications for overall well-being (Welzel and Inglehart, 2010).

In recent decades, a growing number of scholars and professionals have recognized the pivotal importance of SoA. Investigations into the significance of SoA span various domains, including Human-Computer Interaction (Coyle et al., 2012; Togawa et al., 2024), autonomous-driving vehicles (Wen et al., 2019; Yun et al., 2019), developmental robotics (Tani, 2009; Schillaci et al., 2016), user modeling and adaptation (Legaspi et al., 2022), and artificial intelligence (Legaspi et al., 2024).

### 1.2 Measures of SoA

Given its profound influence on human cognition and behavior, the need for reliable and comprehensive measurements of SoA is paramount in both academic research and industrial applications. Cognitive and psychological experiments use indirect and direct methods to measure implicit and explicit SoA, respectively.

There are three types of major indirect measures of SoA, namely intentional binding, sensory attenuation, and visual attention. Although the experimental procedures and calculations vary across each other, the three methods adhere to a consistent principle: they involve measuring the difference in perception and behavior between conditions when people feel a strong sense of agency versus a weak one (see Wen and Imamizu, 2022 for noteworthy references). For example, the basic concept of intentional binding refers to the perceived compression of the time interval between an intentional action and its outcome, compared to an involuntary action (Moore and Obhi, 2012). Intentional binding has been regarded as one of the most promising methods used to measure SoA (Haggard and Tsakiris, 2009) and its relationship to SoA has been reproduced in numerous studies (*cf.* Wen and Imamizu, 2022). However, it still has limitations as a comprehensive SoA measure as some researchers found that the intentional binding effect is sometimes unrelated to explicit measures of SoA (e.g., the direct measure of local SoA “How much do you feel you are in control of the event at this moment?,” see Wen et al., 2015; Majchrowicz and Wierzchoń, 2018; Legaspi et al., 2022). Moreover, Desantis et al. (2011) and Berberian et al. (2012) argue that indirect measures are sensitive to experimental manipulations of people’s beliefs about their agency, and therefore, they are suitable to be used as measures of local and task related SoA rather than decontextualized, chronic, and cross-situational experience of agency.

The general aspect of SoA is measured directly using the Sense of Agency Scale (SoAS). A widely citated SoAS was developed by Tapal et al. (2017). Tapal’s SoAS distinguishes between Sense of Positive Agency (SoPA) and Sense of Negative Agency (SoNA). The former refers to positive feeling in physical and mental control, and its impact on the external environment (“I am in full control of what I do.”), while the latter represents controlless feeling of one’s body and spirit, and its consequential impact (e.g., “My action just happened without my intention.”) In the original paper, the 13-item Likert-scale of SoAS reached appropriate reliability and validity. At the time of preparing this manuscript, Tapal et al.’ paper has reached 188 citations. Its French adaptation (Hurault et al., 2020) and German adaptation (Bart et al., 2023) have also replicated the two-factor structure with acceptable reliability, construct validity and construct stability.

### 1.3 Research problem

Tapal et al.’s SoAS (hereafter, Tapal-SoAS) has also been frequently applied in experiments conducted with Japanese speaking participants. A Japanese translation of Tapal-SoAS has been used by Togawa et al. (2024) and Legaspi et al. (2024) to measure the general SoA of Japanese population in lab environments, and by Legaspi et al. (2022) in complex, natural settings. Although internal consistency of SoPA and SoNA factors reached acceptable level in all these three studies, they did not conduct exploratory nor confirmatory factor analysis to examine whether the two-factor structure of SoAS could be applied in the Japanese context. Thus, it is uncertain whether Tapal-SoAS has a cross-cultural validity in the Japanese context, or whether the factor structure varies from the original one thereby reflecting Japanese geographic and cultural originalities.

We should mention that another SoA scale was developed by Asai et al. (2009; hereafter, Asai-SoAS). Asai and colleagues defined SoA as the self-consciousness whether an action is caused by self or others. The Asai-SoAS involves three factors, namely, “misattribution of agency in one’s mental state” (e.g., “In a crowd, there are moments that I suddenly turn around because I feel someone called my name.”), “lack of control of one’s physical activities” (e.g., “I am occasionally asked to repeat what I said because I failed to control the volume of my voice.”), and “self-appeal in one’s social activities” (e.g., “At times, I prioritize asserting my own opinions over cooperating with others around me.”) While the concepts of Tapal-SoAS and Asai-SoAS have some overlaps, the objectives and use cases are different. Asai-SoAS was developed as a diagnostic tool for schizophrenia, hence, the items were mainly generated based on mental disease scales, such as the Oxford Schizotypal Personality Scale (Claridge and Broks, 1984), Oxford-Liverpool Inventory of Fellings and Experiences (Mason et al., 1995), and Schizotypal Personality Questionnaire (Raine, 1991). In contrast, Tapal-SoAS was developed to measure a general SoA, thus, the applicable population extends beyond individuals that potentially have mental disorders. From this vantage point, our work could be regarded as the first scale measuring the SoA of mentally healthy Japanese individuals.

### 1.4 Present study

The aim of the present study is to provide and validate the Japanese version of Tapal-SoAS (J-SoAS henceforth). We conducted multiple sets of exploratory and confirmatory factor analyses to thoroughly examine the two-factor structure of Tapal-SoAS using a longitudinal survey dataset. Subsequently, we assessed the reliability and construct validity of this adapted scale, alongside investigating construct stability across various age groups, ranging from individuals in their 20s to those in their 60s.

## 2 Materials and methods

### 2.1 Translation and adaptation

Similar to the French adaptation, we strictly followed the procedures for back translation and adaptation proposed by the American Psychological Association and related organizations (i.e., American Education Research Association and National Council on Measurement in Education). Five scholars on psychology, cognitive science, and human-centered computer science participated in the translation and adaptation work. First, two Japanese and English bilinguals translated the items into Japanese independently. Subsequently, they discussed on the differences and reached an agreement on the initial Japanese version of the 13-item list. Then, two more bilinguals translated the Japanese items back to English independently. They also carefully discussed the differences and finally reached an agreement. A native English speaker compared the original English items and the back-translated ones to check whether there are any significant differences on both the content and nuance between the two 13-item lists. The final Japanese version was decided by the five scholars aiming at maintaining Japanese linguistic conventions while keeping the semantic and conceptual similarity to the original scale.

### 2.2 Participants

We conducted a three-round longitudinal online survey from mid-January to mid-February 2023. Initially, we recruited a total of 9,631 participants (with 4,938 identifying as female and 4,693 as male), with age ranging from their 20s to 60s, and all residing in the Tokyo metropolitan area, to participate in the first-round survey. Following a two-week interval, we randomly selected 1,600 participants from those who had successfully completed the first-round survey and sent out the second-round survey. From this selected group, we received responses from 1,448 participants. Subsequently, after another 2 weeks, we distributed the third-round survey to all 1,448 participants who had completed the second-round survey. We collected responses from 1,341 participants this time around.

To identify and exclude participants who may have answered the survey hastily or without due consideration, we included a dummy item in each survey. An example of dummy item is “Please select the item on the right.” Participants who did not pass these attention checks were excluded from the subsequent analyses.

**Figure 1:**
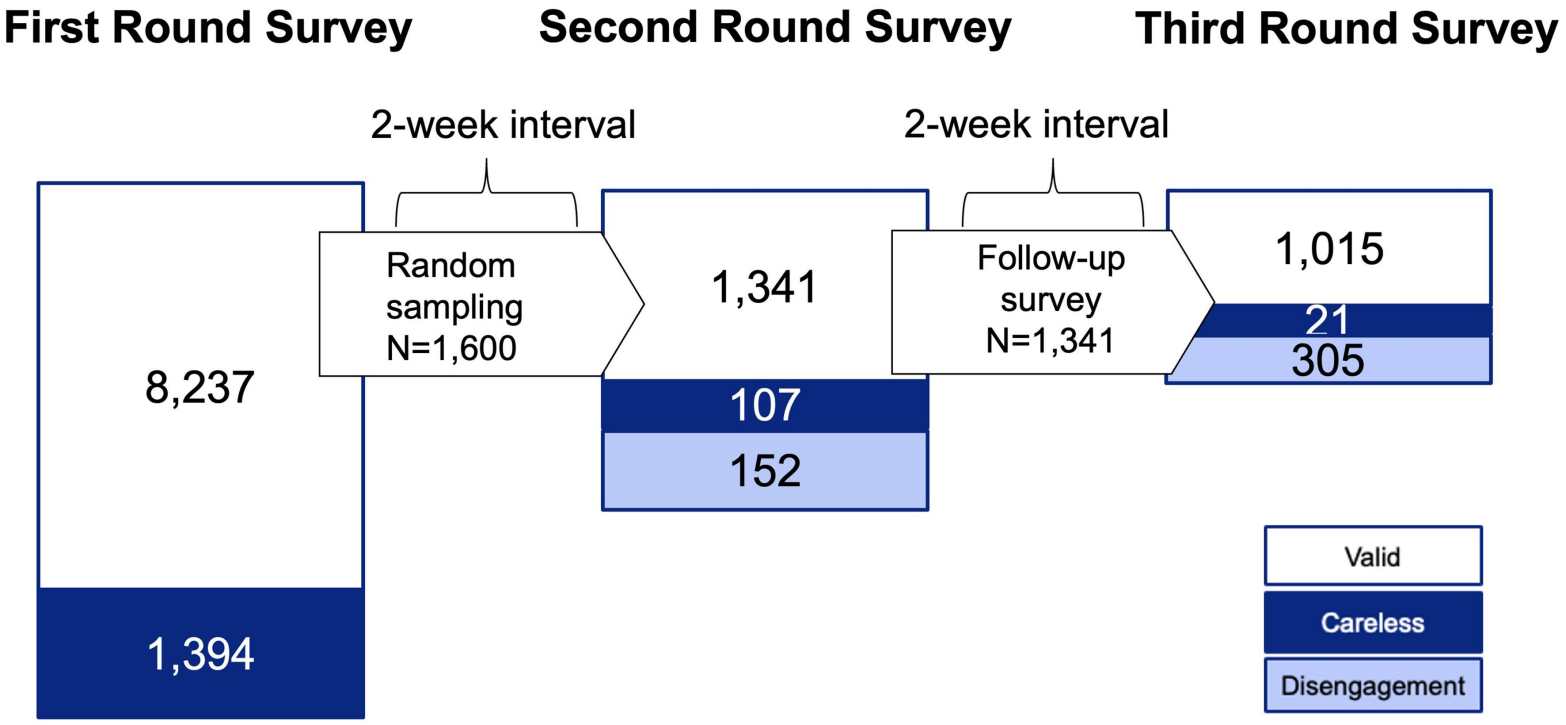
Timetable and participants’ number of the longitudinal survey.

Finally, the sample sizes utilized for statistical analyses in the three rounds of the survey were 8,237, 1,341, and 1,015, respectively. A graphical representation of the timeline and sample sizes is provided in Figure 1. Navy highlights show participants who answered carelessly. Light blue shows those who left the survey.

Throughout the entire process of data collection and analysis, we adhered to the key ethical principles outlined by Bell and Bryman (2007) to diligently protect the dignity, autonomy, and privacy of all participants. Ethical approval was obtained from the institution affiliated with one of the authors.

### 2.3 Measures

To examine the construct validity of the J-SoA, we also collected data on the following relevant constructs. To maintain consistency with the original investigation by Tapal et al. (2017), we administered the Japanese versions of the same instruments. Additionally, we included another Japanese version Sense of Agency scale developed by Asai et al. (2009, referred to as Asai-SoA), to determine whether the newly developed J-SoA and the Asai-SoA measure the same concept.

#### 2.3.1 Asai et al.’ sense of agency

The Asai-SoA measures the self-consciousness aspect of SoA with three factors and 17 items. It is widely used as a diagnostic tool for schizophrenia in psychiatry. All the three factors reached acceptable internal consistency ( $\alpha_{mental}\;$ = 0.72, $\alpha_{physical}\;$ = 0.68, and $\alpha_{social}\;$ = 0.68).

#### 2.3.2 General self-efficacy

GSE represents a generalized, positive belief in personal competence and ability to initiate and execute desired outcome. The general beliefs in self-efficacy have been shown to be potent predictors of “ego strength” and beliefs in personal control (Sherer et al., 1982). In the present study, we applied a well-cited Japanese version of Sherer et al.’s self-efficacy scale developed by Narida et al. (1995). The GSE is comprised of one factor and 23 items across sex and age groups. It reached satisfactory internal consistency ( α = 0.88) and test–retest reliability (*r* = 0.73).

#### 2.3.3 Physical self-efficacy

PSE is a two-factor scale measuring one’s belief in perceived physical ability and self-presentation confidence (Ryckman et al., 1982). The reason we include the PSE is that the perceived control of one’s body is considered as an important aspect of one’s SoA (Elsner and Aschersleben, 2003). We applied a Japanese version of Ryckman *et al*’ PSE that was developed by Matsuo et al. (1999). The 17-item scale replicated the two factor-structure with acceptable internal consistency ( $\alpha_{ability}$ = 0.84 and $\alpha_{confidence}\;$ = 0.71) and test–retest reliability ( $r_{ability}$ = 0.77, $r_{confidence}\;$ = 0.60).

#### 2.3.4 Locus of control

LoC represents one’s belief of control on achieving desired outcome of events in his/her life (Rotter, 1966). The measure developed by Rotter comprises two factors corresponding to the internal and external aspects of LoC. We administered the Japanese adaptation of the LoC scale developed by Kamahara et al. (1982). The Japanese version excluded items related to social and political effects on one’s sense of control. The 18 items that remained were divided into two factors representing Internal and external LoCs, respectively ( $r_{LOC}$ = 0.78).

#### 2.3.5 Free will and determinism beliefs

FAD-Plus is a scale measuring one’s belief about free will (Paulhus and Carey, 2011). It consists of four factors named as Free Will (FW), Fatalistic Determinism (FD), Scientific Determinism (SD), and Unpredictability (UP). We applied the Japanese version of the FAD-Plus scale that was developed by Watanabe et al. (2014). The Japanese version did not include 10 items that have cross-loading and those that were ambiguous to the Japanese participants. As a result, the internal consistency of each factor significantly increased compared to the original scale with no changes in the four-factor structure. The internal consistencies of all the four factors are above 0.70.

### 2.4 Statistical analyses

In the present study, we generally followed the manner of analysis in the original SoAS development paper (Tapal et al., 2017) and its French adaptation (Hurault et al., 2020) to guarantee methodological consistency. As mentioned above, we conducted a three-round longitudinal questionnaire survey. The data of the first-round (*N* = 8,237) was used to perform explanatory factor analysis (EFA) and examine the internal consistency of each factor and the construct stability of the J-SoAS across age. The second-round data (*N* = 1,320) was used to perform confirmatory factor analysis (CFA), and the third (*N* = 1,015) to examine the construct validity. The test–retest reliability was examined in all three sets of data. We performed all statistical analyses in R.

#### 2.4.1 Exploratory factor analyses

Before performing EFA, we first examined the suitability for structure detection using Kaiser-Meyer-Olkin (KMO) test (Kaiser, 1974) of sampling adequacy and Bartlett’s test (Bartlett, 1937) of sphericity. Afterwards, we examined the skewness and kurtosis using Mardia’s test (Mardia, 1970). The KMO measure of sampling adequacy is a statistic that indicates the proportion of variance in the variables that might be caused by underlying factors. It ranges from 0.0 to 1.0, in which a high KMO (close to 1.0) generally indicates distinct and reliable factors could be generated by the data. The minimum acceptable limit of KMO statistic is 0.50 (Field, 2009). The Bartlett’s test of sphericity is used to examine whether the variables in the correlation matrix are correlated. If the *p*-value reaches statistical significance, it indicates that the variables for factor analysis are sufficiently related and therefore suitable for structure detection (Snedecor and Cochran, 1989). Finally, the Mardia’s test examines the normality of the sample data. If the p-value is tested as statistically significant, we admit the data has a deviation from normality. In such case, we apply the principle axis factor, rather than the maximum likelihood, as the appropriate estimator (Fabrigar et al., 1999).

To determine the number of factors, we performed both parallel analysis and minimum average partial test. The former determines the suitable factor solution based on the eigenvalue of the actual data being higher than their corresponding random eigenvalue (Horn, 1965), while the latter focuses on the common variance in a correlation matrix (Velicer, 1976).

We assumed the underlying factors are correlated based on insights of previous studies, hence, we performed EFA with Promax rotation. To examine whether the factor structure vary across different age groups, we repeated the same procedure five times examining the factor structures of ages 20s to 60s.

#### 2.4.2 Confirmatory factor analysis

We conducted CFA using the second-round survey data (*N* = 1,320). We applied the following criteria to evaluate model fit: (1) Root Mean Square Error of Approximation (RMSEA) ≤ 0.10, (2) Standardized Root Mean Square Residual (SRMR) ≤ 0.08, and (3) Comparative Fit Index (CFI) and Tucker–Lewis Index (TLI) ≥ 0.90 (Bollen, 1989).

#### 2.4.3 Reliability, construct validity and construct stability

We computed Cronbach’s alpha (Cronbach, 1951) to examine the internal consistency reliability of each factor using the first-round data. We evaluated the test–retest reliability using the intra-class correlation coefficient (ICC), which proved to be much more efficient due to its sensitivity to detect systematic error (Yen and Lo, 2002). The ICC ranges between 0 and 1, in which values are classified as having poor reliability (ICC < 0.5), moderate reliability (0.5 ≤ ICC < 0.75), and good reliability (ICC ≥ 0.75; Koo and Li, 2016). We examined the construct validity by the correlations between SoA (including SoPA and SoNA) and multiple relevant constructs mentioned in the Measure section.

## 3 Results

### 3.1 Exploratory factor analysis

For the first-round sample (*N* = 8,237), the KMO measure was calculated to be 0.91. The KMO values for each individual item exceeded the threshold of 0.60, with the lowest KMO value observed at 0.89, indicating a satisfactory level for factor analysis. To mitigate concerns of multicollinearity, we examined the inter-item correlations among the 13 items. The highest correlation coefficient observed was below 0.70, thereby resulting in no items being excluded from the EFA. The Bartlett’s test of sphericity yielded a statistically significant result [χ^2^(78, N = 8,237) = 36758.68, *p* < 0.001], complementing the KMO findings and affirming the adequacy of the correlation matrix for factor analysis. Subsequently, we conducted a series of Mardia tests to assess skewness and kurtosis. The results indicated statistically significant values for skewness (Beta-hat skewness = 9.301, Kappa skewness = 12768.82, *p* < 0.001) and kurtosis (Beta-hat kurtosis = 279.23, Kappa kurtosis = 193.54, *p* < 0.001). Given these results, we proceeded with the EFA utilizing the principal axis factoring method.

**Figure 2:**
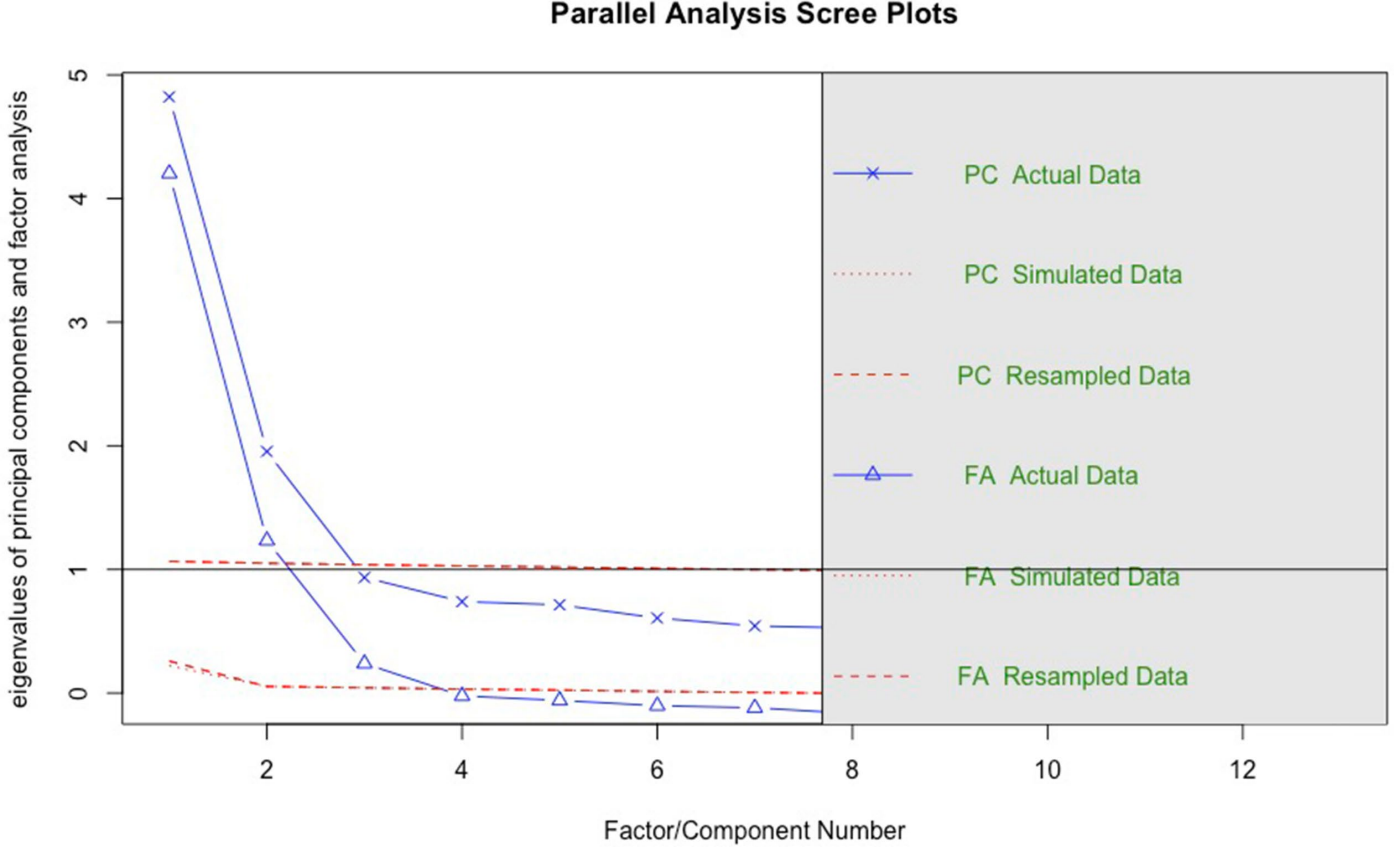
The result of the parallel analysis.

To ascertain the optimal number of factors, two distinct methods, namely parallel analysis and minimum average partial correlation (MAP), were employed. The results of the parallel analysis from EFA indicated a suggestion of three factors and two components (refer to Figure 2). This suggests that while three statistically significant factors were identified in the data, the third factor did not contribute substantially to the variance and could be adequately represented by the first two components.

Simultaneously, the MAP result advocated a two-factor solution, as the Velicer MAP reached a minimum value of 0.02 with 2 factors (Velicer MAP for one to six factor solutions: 0.037, 0.020, 0.027, 0.044, 0.071, 0.104, respectively). Consequently, we concluded that a two-factor solution was most appropriate for our analysis.

Following this determination, Promax rotation was employed on the principal axis factor method. The results revealed that all items exhibited primary loadings exceeding 0.5, with no instances of cross-loading items. As such, the analysis confirmed a 13-item scale, comprising six items within the Sense of Positive Agency (SoPA) factor and seven items within the Sense of Negative Agency (SoNA) factor. The detailed factor structure closely replicated the original scale proposed by Tapal et al.

The correlation coefficient between SoPA and SoNA was found to be −0.44, indicating a moderate negative correlation between the two factors. Table 1 shows the items within each factor.

**Table 1:** Items of the J-SoAS.

SoAS items		SoPA	SoNA
1	I am in full control of what I do	**0.58**	0.04
	私は自分のやることを完全にコントロールできている		
2	I am just an instrument in the hands of somebody or something else	−0.17	**0.69**
	私は他の人の意図や私以外の何かによって操られる装置に過ぎない		
3	My actions just happen without my intention	−0.17	**0.63**
	私の行動は私の意図とは別のところで発生している		
4	I am the author of my actions	**0.71**	−0.10
	私の行動を決めているのは私だ		
5	The consequences of my actions feel like they do not logically follow my actions	−0.04	**0.51**
	私の行動の結果が, 私の行動と論理的に結びついていないように感じる		
6	My movements are automatic—my body simply makes them	0.19	**0.56**
	私は自動的に動いている - 私の体が動きを生み出しているにすぎない		
7	The outcomes of my actions generally surprise me	0.16	**0.54**
	私は基本的に, 自分の行動の結果に驚かされる		
8	Things I do are subject only to my free will	**0.76**	0.10
	私が行ったことは私の自由意志にのみ基づいている		
9	The decision whether and when to act is within my hands	**0.66**	−0.10
	行動するかしないか, いつ行動するか, の判断は私が下す		
10	Nothing I do is actually voluntary	−0.23	**0.53**
	私がすることは実際は自発的なものではない		
11	While I am in action, I feel like I am a remote controlled robot	−0.16	**0.66**
	私は行動している間, 遠隔制御されたロボットのように感じる		
12	My behavior is planned by me from the very beginning to the very end	**0.72**	0.12
	私の行うすべてのことは一番最初から最後の最後まで私自身によって計画されている		
13	I am completely responsible for everything that results from my actions	**0.62**	−0.03
	私は自分の行動の結果の全てに完全に責任がある		
SS Loading/Eigenvalue		**3.06**	**2.61**
Cumulative Variance		**0.44**	**0.24**

The higher of the two loadings appears in bold.

### 3.2 Confirmatory factor analysis

The CFA was conducted utilizing the second-round survey data (*N* = 1,320). Initial testing using the Mardia test revealed statistically significant values for both skewness and kurtosis (Beta-hat skewness = 19.82, Kappa skewness = 4360.22, *p* < 0.001; Beta-hat kurtosis = 303.29, Kappa kurtosis = 99.62, *p* < 0.001). Consequently, the Maximum Likelihood Robust estimator, deemed appropriate in the prior research on the French adaptation of the SoA Scale, was selected (Satorra and Bentler, 1994).

The results of the CFA indicated that the two-factor model provided a good fit to the data: χ^2^(64, *N* = 1,320) = 507.562, *p* < 0.001; CFI = 0.939; TLI = 0.926; RMSEA = 0.072 (90% CI = 0.067, 0.078); SRMR = 0.055. Furthermore, the covariance between factors was found to be *β* = −0.747 (*p* < 0.001), indicating a significant negative correlation.

**Figure 3:**
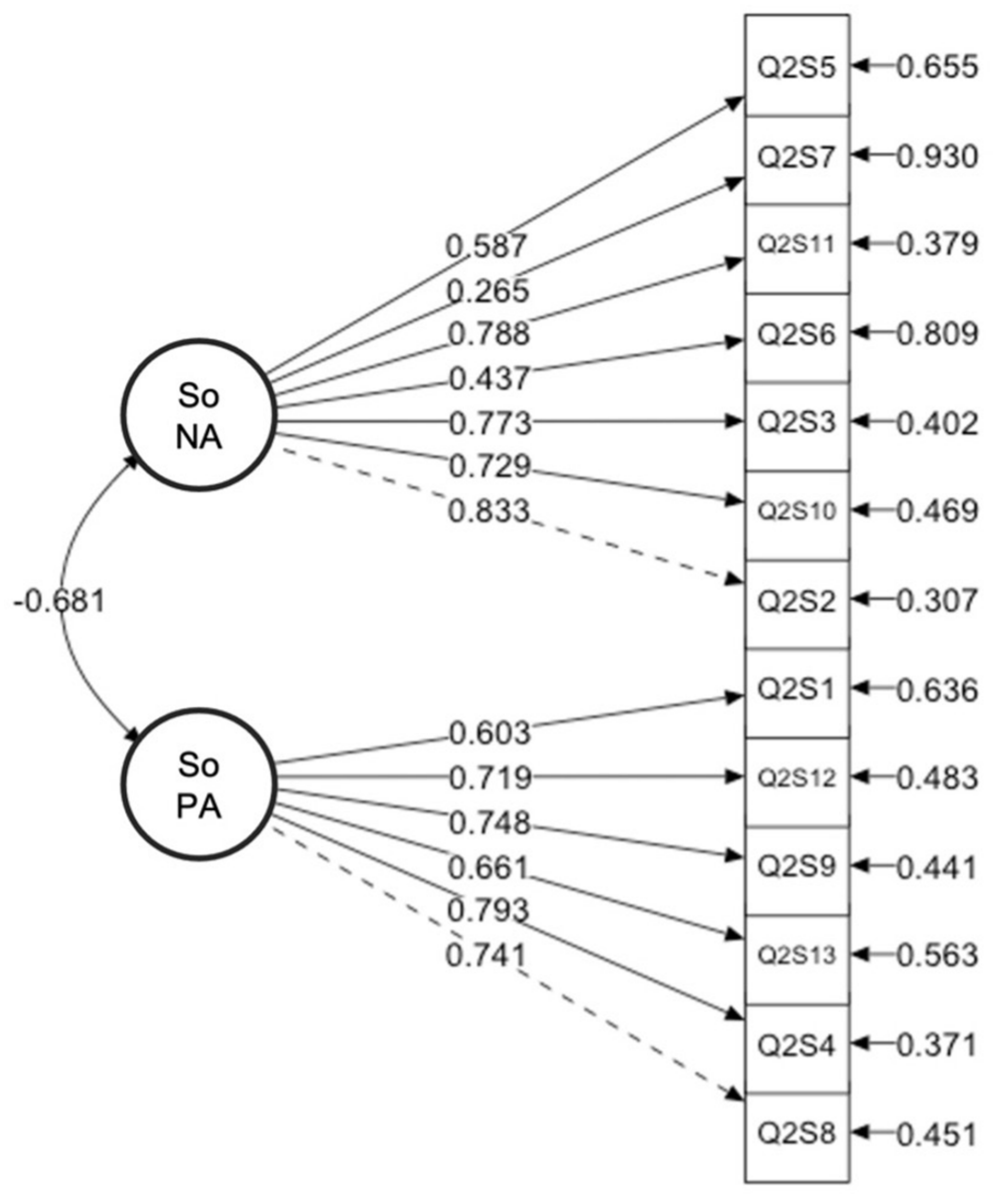
The result of the confirmative factor analysis.

With all fit indices meeting acceptable thresholds, we conclude that the two-factor model provides a satisfactory fit to our data. Figure 3 thoroughly visualizes the model.

### 3.3 Reliability

The internal consistency reliability of the scale was assessed using the first-round data. The estimated Cronbach’s alpha coefficients were 0.839 (95% CI = 0.823, 0.853) for the SoPA factor and 0.801 (95% CI = 0.782, 0.818) for the SoNA factor. These results indicate a satisfactory level of internal consistency reliability for both factors.

To evaluate the test–retest reliability, we computed the intra-class correlation coefficients (ICCs) using SoA scores collected at three different time points. The ICCs were found to be 0.655 (95% CI = 0.591, 0.708) for the SoPA factor and 0.664 (95% CI = 0.626, 0.698) for the SoNA factor. In accordance with the criteria proposed by Koo and Li (2016), both factors demonstrated at least moderate test–retest reliability.

### 3.4 Construct validity

The construct validity of the scale was assessed by examining the correlations between the SoA scores and several relevant constructs using the data from the third-round survey. A detailed summary of the results is provided in Table 2.

**Table 2:** Latent correlations between the SoAS factors and other constructs.

	SoPA	SoNA
GSE	0.39*	−0.33*
PSE-ability	0.15	0.05
PSE-confidence	0.34*	−0.34*
ELOC	−0.30*	0.39*
ILOC	0.48*	0.33*
FAD-free will	0.45*	−0.20*
FAD-fatalistic D	−0.22*	0.36*
FAD-Scientific D	−0.05	0.17
FAD-unpredictability	0.08	−0.05
Asai’s SoA-mental	−0.23*	0.37*
Asai’s SoA-physical	−0.25*	0.36*
Asai’s SoA-social	−0.23*	0.03

SoPA, Sense of Positive Agency; SoNA, Sense of Negative Agency; GSE, General Self-Efficacy; PSE, Physical Self-Efficacy; Fatalistic D, Fatalistic Determinism; Scientific D, Scientific Determinism; Asai’s SoA, Japanese scale of Sense of Agency developed by Asai et al. *Significant at *p* < 0.05.

Overall, the findings were largely consistent with previous investigations conducted by Tapal and colleagues. Specifically, moderate correlations were observed between SoA measures and constructs such as general self-efficacy (GSE), External and Internal locus of control (ELOC and ILOC), and the confidence factor of Personal Self-Efficacy (PSE). Furthermore, the Free Will factor of the FAD-Plus questionnaire exhibited moderate correlations with the SoPA and weak correlations with the SoNA. Conversely, the Fatalistic Determinism factor of the FAD-Plus showed moderate correlations with SoNA and weak correlations with SoPA. These results closely align with those reported in the original scale.

Notably, SoPA demonstrated weak correlations with all three measures of Asai’s SoA, while SoNA exhibited correlations with only a subset of Asai’s SoA measures. This observation suggests potential discrepancies or differences in compatibility between the two sets of measures. It is possible that the measures named “Sense of Agency” are developed to capture distinct aspects of the construct, or they may represent two related yet distinct concepts under the umbrella term of “SoA.”

In this context, the findings underscore the necessity of our current study in providing insights into the understanding and measurement of the multifaceted concept of SoA.

### 3.5 Construct stability

**Figure 4:**
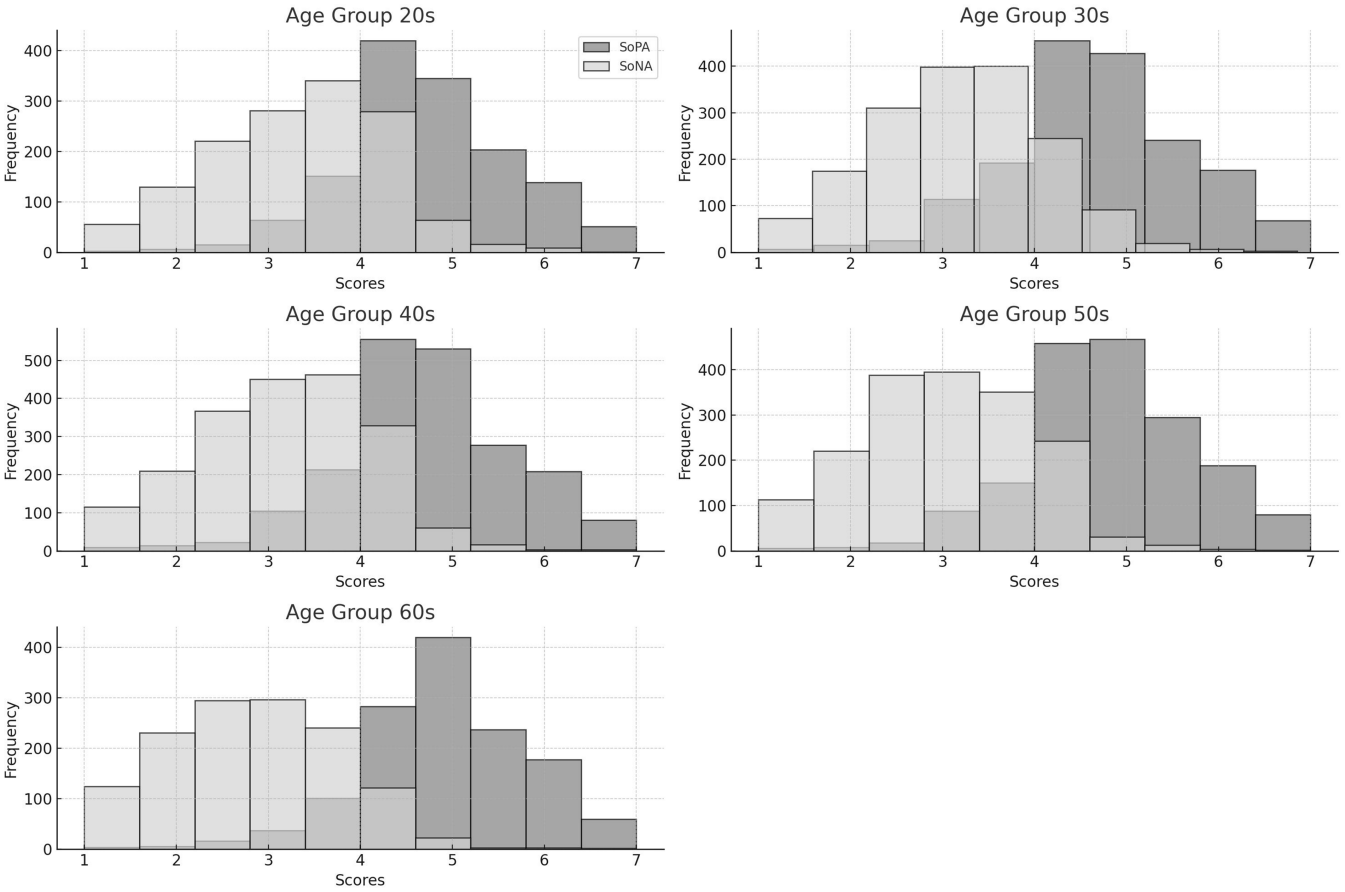
The distributions of SoPA and SoNA scores across age cohorts.

To examine whether the two-factor solution fits well each age groups, we repeated five sets of confirmatory factor analyses that followed the same procedure described in Section 2.6. The results of model fitting the 20s to 60s age groups are shown in Table 3. The acceptable model fit indicated that the two-factor solution is stable across age. The histograms showing the distribution of SoPA and SoNA are presented in Figure 4. The distribution of the SoA scores appear similar across age groups.

**Table 3:** Results of the confirmatory factor analyses of different age groups.

	N	df	χ^2^	*p*	CFI	TLI	RMSEA	SRMR
20s	1,398	64	537.298	< 0.001	0.912	0.893	0.073	0.068
30s	1722	64	718.888	< 0.001	0.909	0.890	0.077	0.064
40s	2015	64	800.639	< 0.001	0.916	0.897	0.076	0.060
50s	1760	64	723.874	< 0.001	0.924	0.907	0.077	0.056
60s	1,342	64	620.198	< 0.001	0.917	0.899	0.080	0.060

## 4 Discussion

In this study, we introduced a Japanese adaptation of Tapal et al.’s Sense of Agency Scale (J-SoAS) and assessed its reliability and validity through extensive large-scale and longitudinal surveys. Additionally, we investigated the construct stability of the J-SoAS across various age cohorts. Our findings provide reliable evidence that the J-SoAS maintains the same two-factor structure as the original scale, demonstrating acceptable reliability and construct validity for assessing general and explicit SoA. Furthermore, our results indicate that this factor structure remains stable across different age groups.

### 4.1 Psychometric findings

We replicated the two-factor structure proposed in the original study using both EFA and CFA. Similar to the original SoAS, the J-SoAS comprises a total of 13 items divided into two factors: SoPA (Sense of Positive Agency) and SoNA (Sense of Negative Agency). All indices related to test–retest reliability, internal consistency, and construct validity achieved acceptable levels.

It should not be ignored that the test–retest reliability only reaches moderate level, which is consistent with the French and German adaptations. One possible explanation is that general SoA, which refers to a broad belief in one’s agency across situations, may be temporarily influenced by contexts. Legaspi et al. (2022) observed this phenomenon in their longitudinal study. They examined the relationship between general SoA and healthy eating intentions and behaviors, measuring general SoA three times over 2 months using an experimental smartphone app. They found that general SoA slightly increased over time, and its impact on goal pursuit (e.g., healthy eating behavior) also grew. They interpreted this as a mutual influence, where high SoA enhances goal-directed behavior, and achieving goals, in turn, boosts SoA.

Our results provide preliminary evidence supporting the cross-cultural validity of the SoAS in measuring SoA among Japanese individuals. Our findings align with the conclusions of Tapal et al. (2017), Hurault et al. (2020) and Bart et al. (2023), suggesting that cultural influences may have limited impact on the fundamental structure of global SoA.

To the best of our knowledge, our study represents the first examination of the construct stability of explicit and general SoA measures across diverse age groups. Recent works have begun exploring the effects of physical aging on one’s experience of agency. It is posited that physiological aging often correlates with a decrease in sensory sensitivity (Boisgontier and Nougier, 2013), which may subsequently influence the SoA. A limited yet sophisticated body of research has observed participant behavior and suggests that elderly individuals may exhibit reduced levels of agency (Metcalfe et al., 2010; Cioffi et al., 2017). Similarly, Mariano et al. (2024) investigated the negative impact of aging on both implicit and explicit local SoA using laboratory experiments.

It is notable that the correlations between SoPA and SoNA vary across different investigations. The French and German adaptations reported moderate correlations between SoPA and SoNA, with values of −0.56 and − 0.65, respectively. These findings are consistent with our result of −0.68. These correlations are higher than the original study’s reported value of −0.38. This discrepancy may be due to differences in the average age of participants across the studies. Tapal et al. (2017) recruited 236 college students with an average age of 24.3 (SD = 3.6), while Bart et al. (2023) used a mixed sample with an average age of 28.5 (SD = 10.5). Our study included participants aged from 20s to 60s.

To explore if the correlation between SoPA and SoNA varies with age, we conducted Pearson correlational analyses for each age group. The correlation coefficients for ages 20s to 60s were − 0.33, −0.37, −0.44, −0.54, and − 0.49, respectively. The correlations for the 20s, 30s and 40s groups align with Tapal et al. (2017), and the 50s and 60s groups align with Bart et al. (2023). We excluded the French study’s results because they omitted six items from the original scale.

We acknowledge that multiple factors may contribute to these differences. However, due to limited resources, we cannot investigate potential cultural differences at this time. To avoid over-interpretation, we suggest this as an open question for future research.

In this study, our large and diverse sample, spanning a wide range of age, enabled us to explore the cross-age validity of a comprehensive explicit and general SoA scale. As demonstrated in Table 3, all two-factor models across different age groups achieved acceptable model fit. The structural equivalence of the SoAS facilitates direct comparisons across various age demographics.

### 4.2 Limitations and future plans

Our study confirmed the stability of the two-factor structure of the J-SoAS across participants in the 20 to 60 age range. This provides initial evidence for the applicability of the J-SoAS in cross-age comparisons. However, for more stringent applications, such as assessing one’s mental state, a more sophisticated statistical approach should be considered. Future investigations could explore measurement equivalence using multigroup simultaneous analysis. This method would allow for a more rigorous examination of the scale’s consistency across different age cohorts and ensure its suitability for precise psychological assessments.

Second, our study lacks representation from individuals under 20 and above 70 years old. This limitation prevents definitive conclusions regarding the adaptability of the two-factor structure for very young children, teenagers, and the elderly over 70. To address this gap, future research should aim to include participants from a broader age spectrum. This inclusive approach would facilitate a comprehensive examination of the J-SoAS’ validity and applicability across different developmental stages and life phases.

Third, our study also drew comparisons with the French adaptation of the SoAS, as conducted by Hurault et al. (2020). Like our findings, the French adaptation replicated the two-factor structure of Tapal et al.’s Hebrew version of the SoAS. This suggests that there may be little impact of culture on the general SoA. However, it is important to note that the French model reported low construct validity after the removal of six unsuitable items, while our model retained all 13 items proposed by the original SoAS. This discrepancy raises the possibility that the two-factor structural scale with 13 items fits the Hebrew and Japanese populations better than it does the French population. Therefore, it is worthwhile for subsequent studies to investigate cross-cultural differences in the adaptation of the SoAS in a more sophisticated manner. This could involve detailed analyses of item response patterns, differential item functioning across cultures, and the cultural nuance of specific scale items.

## 5 Conclusion

In conclusion, this study has sought to replicate the two-factor structural measure of general and explicit SoA within the Japanese context. The results indicate that the J-SoAS exhibits acceptable levels of reliability, construct and cross-cultural validity, as well as structural stability across diverse age groups. This adaptation offers a potential contribution toward a deeper understanding of SoA concepts and presents a reliable instrument for quantitative investigations on SoA among the Japanese population.

Furthermore, the J-SoAS holds promise for facilitating comparisons of general and explicit SoA between various cultural groups, such as those from Western and Eastern backgrounds, as well as across different age demographics including youth and the seniors. Such comparisons could provide valuable insights into the nuanced nature of SoA across cultural and generational divides.

In essence, the development of the J-SoAS represents a step forward in providing researchers with a reliable and valid tool for exploring general and explicit SoA. Its potential applications may serve to accelerate research efforts and expand our understanding of SoA within a range of disciplines and contexts.

## Data availability statement

The data that support the findings of this study are available from the corresponding author, WX, upon reasonable request. The data is only for educational and academic use.

## Ethics statement

The studies involving humans were approved by Research Ethics committee of KDDI research, Inc. The studies were conducted in accordance with the local legislation and institutional requirements. The ethics committee/institutional review board waived the requirement of written informed consent for participation from the participants or the participants’ legal guardians/next of kin because data for our study was meticulously collected via an online survey platform, ensuring ethical standards. Only participants who reviewed and consented to the informed consent form were allowed to proceed with the survey, guaranteeing that all responses were gathered with full awareness and voluntary participation.

## Author contributions

WX: Conceptualization, Formal analysis, Funding acquisition, Investigation, Methodology, Validation, Writing – original draft, Writing – review & editing. RL: Conceptualization, Investigation, Writing – review & editing. YI: Conceptualization, Writing – review & editing, Investigation. YW: Writing – review & editing.
